# Isolating the role of the matrix at patch and landscape scales

**DOI:** 10.1111/1365-2656.70089

**Published:** 2025-06-24

**Authors:** Thomas A. H. Smith, Robert D. Holt, Emilio M. Bruna, Robert J. Fletcher

**Affiliations:** ^1^ Department of Wildlife Ecology and Conservation University of Florida Gainesville Florida USA; ^2^ Department of Integrative Biology University of Wisconsin‐Madison Madison Florida USA; ^3^ Department of Biology University of Florida Gainesville Florida USA; ^4^ Center for Latin American Studies University of Florida Gainesville Florida USA; ^5^ Department of Zoology Conservation Research Institute, University of Cambridge Cambridge UK

**Keywords:** edge effects, fragmentation, habitat composition, landscape experiment, landscape matrix, spatial scale

## Abstract

1. Global increases in habitat loss and fragmentation have resulted in non‐habitat landcover, or the matrix, becoming an increasingly prominent feature of landscapes. The matrix can influence the population dynamics of species in fragments by modifying processes operating locally on individual patches (e.g. edge effects on survival) or at landscape scales (e.g. inter‐patch dispersal). However, the relative magnitude of patch‐ vs. landscape‐scale matrix effects on the populations found in patches remains unclear.
2. We established 12 experimental landscapes in which we controlled for habitat amount and fragmentation while manipulating the quality of the matrix around (i) individual habitat patches and (ii) across the entire landscape in a factorial design. We then compared the magnitude of local‐ and landscape‐scale matrix effects on a specialist herbivore, *Chelinidea vittiger* (Hemiptera: Coreidae).
3. Population size in fragments was influenced by both patch‐ and landscape‐scale treatments: Population size increased in patches surrounded by high‐quality matrix, but only in landscapes dominated by low‐quality matrix, due in part to decreased inter‐patch movements in these landscapes. In contrast, the effects on both survival and reproductive output were solely at the patch‐scale, with both lower in patches surrounded by low‐quality matrix.
4. Our results underscore the outsized importance of matrix habitat immediately adjacent to fragment edges—despite the fact that patch‐scale manipulations affected only a fraction (3%) of the area that landscape‐scale manipulations did, patch‐scale effects were more common. The relationship between dispersal, population size and scale‐dependent effects of matrix quality emphasizes the need to explicitly consider the spatial scale at which different processes operate when predicting responses to habitat fragmentation. Our results also suggest the matrix immediately adjacent to habitat remnants is of particular importance when considering alternative strategies for landscape conservation or restoration.

## Graphical abstract

This is the first experimental test of the spatial scales of matrix effects. Both patch‐scale and landscape‐scale matrix influence populations through a cross‐scale interaction altering survival, reproduction, and movement. Conservation in fragmentated landscapes should consider the matrix context of patches while focusing on local matrix due to its outsized influence.

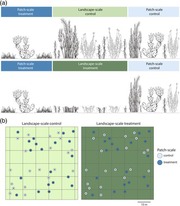

## 1 INTRODUCTION

The type and arrangement of land surrounding habitat can influence a wide variety of ecological and evolutionary processes. As habitat loss and anthropogenic disturbance continue to shape ecosystems, the non‐habitat ‘matrix’ (i.e. land use and/or environmental conditions that differ from either species habitat or reference conditions; Fletcher et al., 2024) in which remnant patches of habitat are embedded is increasingly important for species. The matrix can alter species abundance, dispersal, patterns of gene flow and the distribution of resources on which species depend (DiLeo & Wagner, 2016; Prugh et al., 2008; Watling et al., 2011). When evaluating landscapes using a species‐centric delineation of the matrix, evidence that both the type of matrix and its structural similarity to habitat can determine ‘matrix quality’, affecting the survival and reproduction of individuals with consequences for both population structure and metapopulation dynamics (Prevedello & Vieira, 2010). For instance, the quality of matrix surrounding habitat patches can influence the density and emigration rate of individuals (Haynes, Diekötter, & Crist, 2007), which can lead to changes in patch occupancy rates (Haynes, Dillemuth, et al., 2007). However, the underlying mechanisms responsible for these patterns often remain unclear (Driscoll et al., 2013; Ruffell & Didham, 2016).

The matrix of a landscape can influence animals persisting in habitat fragments through processes operating at two fundamentally different spatial scales: the local patch scale and the landscape scale (Fletcher et al., 2007; Prevedello & Vieira, 2010). Patch‐scale effects occur when the type of matrix surrounding an individual fragment alters the resident population's demographic rates. These local effects, often conceptualized as ‘edge type’ or ‘edge contrast’ effects, alter the quality and resource availability at the patch‐matrix edge (Ries et al., 2004). Edge effects can operate through biotic (e.g. predation) and abiotic (e.g. microclimate) spillover from the matrix into adjacent habitat (Ries & Sisk, 2004; van Schalkwyk et al., 2020), supplementation with similar resources, complementation from novel resources in the surrounding matrix (Haynes, Diekötter, & Crist, 2007; Ouin et al., 2004) or by altering conditions influencing dispersal decisions (Haynes & Cronin, 2006). In contrast, landscape‐scale matrix effects influence populations primarily by altering outcomes of inter‐patch dispersal. For instance, the matrix can alter the likelihood of movement between patches (Prevedello & Vieira, 2010; Ricketts, 2001) or the survival of dispersing individuals (Fletcher et al., 2019; Revilla & Wiegand, 2008; Schwab & Zandbergen, 2011). However, isolating effects from these two distinct scales of matrix effects on populations remains limited (Kuefler et al., 2010).

Despite the widespread view that matrix effects are pervasive, most support currently comes from observational studies (Prevedello & Vieira, 2010; Ramírez‐Delgado et al., 2022; Watling et al., 2011), limiting our understanding of the mechanisms driving matrix effects. Fletcher et al. (2024) reviewed 2048 articles related to matrix effects, finding only 39 that manipulated matrix conditions. While there was clear evidence from these experiments that the matrix had widespread effects, none of these experiments manipulated the matrix at both the patch and landscape scales. Since these scales have yet to be disentangled, the relative importance of each scale is also unclear. Furthermore, cross‐scale interactions of matrix effects may occur but have not been isolated.

Interaction between the two scales of the matrix can occur if aspects of the matrix directly surrounding a patch alter the number of individuals experiencing the effects of the matrix across the landscape. For instance, matrix conditions at the patch edge that increase rates of emigration from patches (Haynes & Cronin, 2006; Haynes, Dillemuth, et al., 2007) should increase the influence of landscape‐scale matrix variation as more individuals in the population traverse the landscape. In contrast, matrix conditions at the patch edge that increase the ability of individuals to find preferred habitat could reduce the time spent moving through the matrix, and thus decrease its overall influence. Similarly, landscape‐scale matrix conditions that increase dispersal success either through increased permeability or survival allow more individuals to relocate and experience variation in local patch conditions arising from patch‐scale matrix effects (Matthews, 2021). The consequences of this potential cross‐scale interaction of behavioural and demographic effects would depend on the relative intensity of matrix effects operating at both scales and which population parameters (e.g. dispersal, survival or reproduction) are affected. Such potential effects can be challenging to characterize in purely observational studies. A landscape experiment manipulating each scale independently and jointly is necessary to fully disentangle each impact of the matrix and determine the mechanisms driving population dynamics in fragmented landscapes.

We tested for matrix effects operating at two scales and elucidate the demographic changes driving these effects using a landscape experiment on a specialist insect herbivore, *Chelinidea vittiger* (Hemiptera: Coreidae), and its patchy habitat, *Opuntia mesacantha* (Caryophyllales: Cactaceae). Higher vegetation height of the landscape in which *Opuntia* is embedded has previously been shown to increase survival of *C. vittiger* in the matrix and accounting for this aspect of the matrix improves predictions of movement and population size (Fletcher et al., 2019). While movement rates were decreased in higher matrix when considered at a fine scale (1–2 times the perceptual range; Fletcher et al., 2014; Schooley & Wiens, 2004), realized patch immigration rates increased with higher matrix heights (Acevedo & Fletcher, 2017). Following the expectation that species move less quickly though high‐quality areas (Crone et al., 2019), we consider matrix areas with high vegetation height as high‐quality matrix and areas with low vegetation height as low‐quality matrix. Therefore, experimentally reducing the vegetation height of a landscape provides a straightforward means to reduce matrix quality for this species. Across 12 landscapes, we manipulated the matrix by reducing the height of vegetation at both the patch scale directly surrounding patches and across the entire landscape. We then address the following questions. First, what are the relative effects of variation in the matrix at patch and landscape scales on per‐patch population abundance and do cross‐scale interactions of these matrix effects occur? Second, how are the demographic parameters of adult survival, reproductive output measured as nymph abundance, and dispersal probability influenced by variation in the matrix at these two scales?

## 2 MATERIALS AND METHODS

### 2.1 Study area and experimental system

We conducted this experiment at Ordway Swisher Biological Station (OSBS; 29.4 N, 82.0 W) located in north‐central Florida, USA. We focussed on the cactus bug (*Chelinidea vittiger*), which specializes on *Opuntia* cactus. The *C. vittiger* and *Opuntia* system is a model mesocosm for experimentally studying habitat fragmentation and landscape processes at realistic spatial scales (Fletcher et al., 2018; Fletcher, Revell, et al., 2013; Schooley & Wiens, 2004, 2005). In our study area, *C. vittiger* is a specialist that lives and feeds on the dwarf prickly pear *Opuntia mesacantha*, which is patchily distributed in old fields. Nymphs of *C. vittiger* rarely leave the *Opuntia* on which they hatched (Schooley & Wiens, 2004), and while adult *C. vittiger* have the ability to fly, they rarely do (DeVol & Goeden, 1973; Mann, 1969). Those *C. vittiger* individuals that do move from one *Opuntia* patch to another typically do so by walking through the intervening non‐habitat matrix (DeVol & Goeden, 1973; Schooley & Wiens, 2004). The naturally patchy distribution of *Opuntia*, coupled with *C. vittiger*'s patch fidelity, life‐history and dispersal mechanism, makes this system ideally suited to experimentally assessing the effects of fragmentation on habitat specialists (Fletcher et al., 2018, 2023).

Matrix vegetation surrounding patches of *Opuntia* in OSBS consists of graminoids, such as wiregrass (*Aristida stricta*) and broom sedge (*Andropogon spp*), forbs such as dogfennel (*Eupatorium capillifolium*) and narrow‐leaf silk grass (*Pityopsis graminifolia*), and scattered woody species consisting of gopher apple (*Geobalanus oblongifolius*), pawpaw (*Asimina incana*) and brambles (*Rhubus* sp.). The resulting matrix for *Opuntia* habitat is highly variable. The height of this matrix is known to affect the movement rates of *C. vittiger* in this system both at OSBS (Acevedo & Fletcher, 2017; Fletcher et al., 2014) and elsewhere (Schooley & Wiens, 2004).

In north‐central Florida, *C. vittiger* is reproductively active from March through September, with females laying eggs directly on *Opuntia*. Upon eggs hatching, there are five nymphal instars, with adult emergence occurring after approximately 53 days (Mann, 1969). This results in 2–3 generations per year, with individuals overwintering as adults. Nymphs typically remain at the oviposition site such that nymph abundances correspond to the adult reproductive output of a patch (Miller et al., 2013).

### 2.2 Experimental design

We constructed 12 50 m × 50 m landscape plots that were paired into six geographic blocks, with at least 35 m separating landscapes within a pair. Much larger distances (>150 m) separated geographic blocks (i.e. paired landscapes). This landscape size is based on known movements of cactus bugs (Fletcher et al., 2011, 2018), and this size has been used elsewhere as a relevant landscape extent for this plant–herbivore system (Fletcher et al., 2018, 2023) with the separation of 35 m ensuring that there is negligible inter‐landscape dispersal (only 2 out of 40 observed dispersal events in this experiment occurred between landscapes). Landscape locations were selected where *Opuntia* occurs naturally. All cactus within each landscape and a 5‐m buffer surrounding a landscape was completely removed. After 2 weeks, cactus was transplanted from nearby wild populations to create habitat patches. All naturally occurring cactus bugs were removed from cactus used for transplantation. Transplantation occurred at least 1 month prior to experimental treatments described below to allow for cactus to establish. Cactus patches were reinspected for cactus bugs prior to initialization of the experiment and any bugs detected were removed to ensure no natural cactus bugs remained in the landscape. All landscapes were constructed with permission from OSBS, and this experiment did not require ethical approval.

We planted patches in each landscape at locations determined from realizations of a Thomas cluster point process (Baddeley et al., 2015) fit to data of natural distributions in the study area (Fletcher et al., 2023). Thomas cluster point processes are generative models that can create clustered distributions of points; these types of processes are commonly used in plant ecology to understand and model the dispersion of plants, particularly in situations where dispersal is limited (e.g. Lin et al., 2011). Clustering is modelled using ‘offspring’ points based on a Gaussian function with the Thomas cluster point process, which captures the naturally patchy cactus distribution resulting from *Opuntia*'s ability to vegetatively reproduce via cladode detachment (Reyes‐Agüero et al., 2006). Patches were at least 2 m apart to allow for patch‐scale matrix treatments (see below) resulting in spatial distributions in which patches had an average nearest neighbour distance of 5.4 meters across all landscapes (median 5.2 m; Figure S2). To focus on matrix conditions while controlling for habitat amount and fragmentation, we planted 40 patches per landscape of equal patch size (based on the number of segments, which is strongly correlated to basal area; *r* = 0.91, Figure S1). Each patch contained 17 cactus segments and had a circular patch area approximately 40 cm in diameter, which was based on the upper 70th percentile of the natural size distribution in the study area (taken from Fletcher et al., 2018). Individuals of *C. vittiger* treat cactus within 50 cm as a single patch (Schooley & Wiens, 2004). This patch definition is highly meaningful in explaining responses to fragmentation for this species at both patch and landscape scales (Fletcher et al., 2023).

**FIGURE 1:**
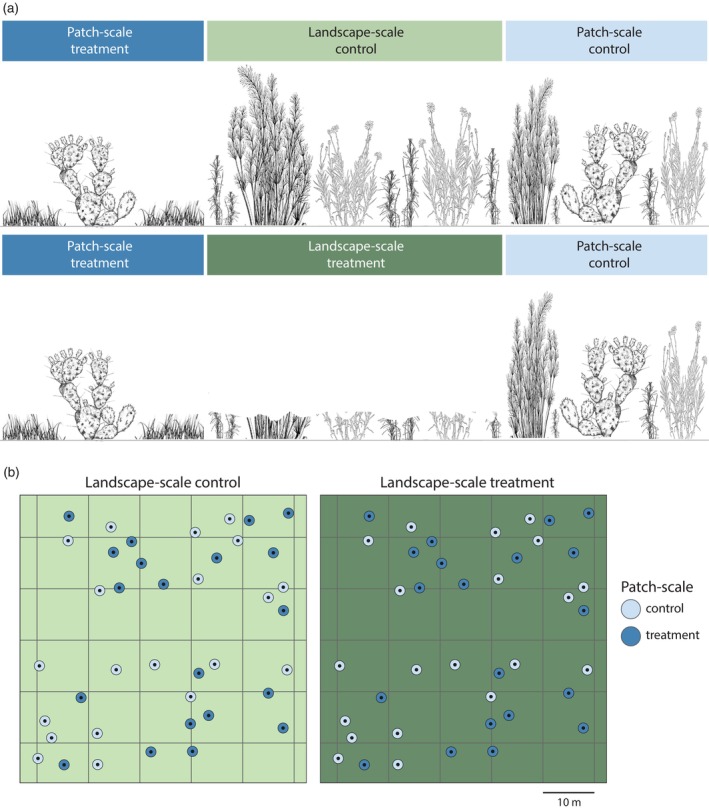
Representational layout for experimental treatments for each landscape pair. (a) Horizontal view from the perspective of study organism for the manipulations of the matrix at the patch‐scale (blue headers) and the landscape‐scale (green headers). (b) Overhead schematic of a pair of 50 m × 50 m landscapes.

To evaluate matrix effects at the local and landscape scales, we used mechanical mowing to reduce the vegetation height of the matrix and in turn reduce matrix quality. We used two treatments: one focussed on patch‐scale and another on landscape‐scale processes. For patch‐scale treatments, we mowed a 1‐m ring of vegetation directly surrounding patches or left the 1‐m ring at its natural height (Figure 1). For this treatment, our aim was to only manipulate conditions immediately surrounding patches. A 1‐m distance is double the distance at which individual *C. vittiger* treat patches as contiguous (50 cm; Schooley & Wiens, 2005) and is at the upper end of the perceptual range of individual *Opuntia* segments by *C. vittiger* (approximately 1 m; Fletcher, Maxwell, et al., 2013). The landscape‐scale treatment of the matrix consisted of mowing the vegetation across an entire landscape except for 1 m directly surrounding cactus patches. We applied treatments using a split‐plot design, with the main plot being the landscape‐scale treatment (mowed v control) and the split‐plot being the patch‐scale treatments, where half of the patches received the mowing treatment around patches (*n* = 20) and the remainder were controls (*n* = 20). Each block included one manipulated landscape‐scale treatment and one control landscape selected at random. Within each block, we constructed the same spatial arrangement of patches for each landscape to control for any possible effects of relative spatial positions (Figure 1b). Patches were chosen at random for patch‐scale treatments and were consistent within a block (Figure 1b: top left patch has the same patch‐scale treatment for both landscapes). This resulted in six total blocks each containing one control and one treatment landscape, with 20 control patches and 20 treatment patches within each landscape.

The experiment was initiated in August of 2019 with 40 adults released at 10 randomly chosen patches (four/patch) in each landscape in the same relative location for landscapes within blocks. This initial population size corresponds to the naturally low occupancy and abundances of *C. vittiger* (Fletcher et al., 2018). Permits were not required for the collection and marking of *C. vittige*r or *Opuntia* used in this experiment. We released two male and female pairs of reproductive age evenly split between patches with the patch‐scale treatment and control to ensure uniform starting abundances and population structure (i.e. sex ratio and age). These bugs were raised in an open‐air greenhouse from wild‐caught parents taken from OSBS in the spring of 2019. We applied treatments 2 weeks prior to release and initiated surveys one month post release to allow for populations to settle and for reproduction to begin. In a prior experiment that included a similar release design (Fletcher et al., 2018), it was shown that population distributions rapidly equilibrated within approximately 1 month post release. Mowing treatments were readministered once every spring and fall for the duration of the experiment to ensure consistent matrix height while limiting disturbance to plots. Each mowing manipulation was performed 1 month prior to the initiation of surveys for that season.

We surveyed the populations 11 times between fall 2019 and spring 2021: three surveys in fall 2019 (Sept–Nov.), one in April 2020 (surveys were limited in spring and summer 2020 due to COVID‐19 restrictions), four in fall of 2020 (Aug–Dec.) and three in spring of 2021 (Apr–June). During each survey, we visited each patch in every landscape. One block was not visited in one survey at the end of 2020 as well as one landscape in 2021. This resulted in a sample size of 5160 patch survey observations from the 480 total patches in the experiment. We counted adult *C. vittiger* to estimate relative population abundance at each patch and counted nymphs as an index of reproductive output. Adults were captured and marked with unique three‐letter codes on their pronotum to allow for standard capture–mark–recapture (CMR) analyses.

### 2.3 Analysis

We tested for differences in patch abundance of adult *C. vittiger* using Zero‐inflated Generalized Linear Mixed Models (GLMMs) with Poisson distributed errors (ZIP; Brooks et al., 2017), including random effects of block, landscape, and year and using package glmmTMB version 1.1.4 in program R version 4.2.1. The inclusion of year accounted for fluctuations in populations due to time and weather conditions over the course of the experiment. The zero‐inflated model corrects for overdispersion caused by the low natural occupancy of cactus patches by *C. vittiger* (across all surveys, naïve patch occupancy = 11.2%). Because of the large number of individual patches (*n* = 480), we did not include an individual random effect (Bolker et al., 2009). Surveys were sufficiently spaced in time to avoid temporal autocorrelation (see Supplemental Materials). We used the DHARMa package (Hartig, 2024) to assess model fit and check for overdispersion in model residuals.

To assess the impact of patch‐ and landscape‐scale matrix treatments on reproductive output, we modelled the patch abundance of nymphs using similar Zero‐inflated GLMMs. Because analysis of the residuals suggested overdispersion with Poisson model (*p* < 0.05) we used a zero‐inflated negative binomial model instead (ZINB; Brooks et al., 2017). We considered a model set that included models with the interaction of both patch‐ and landscape‐scale treatments, the additive effect of each scale only, individual effects of each scale, and a null model with only the random terms included. We used AICc for model selection for all model sets (Anderson & Burnham, 2004; Bolker et al., 2009). Because AICc can lead to the inclusion of variables with little explanatory power, we also used 95% confidence intervals to assess the importance of variables in the top model (Anderson & Burnham, 2004). We used multiple contrasts to assess the magnitude and importance of landscape‐ and patch‐scale matrix effects and their potential interactions using R package emmeans (Version 1.8.1) with Tukey's correction for multiple comparisons.

### 2.4 Disentangling movement and survival processes

We implemented a multi‐state mark–recapture analysis (Lebreton et al., 2009) using Rmark version 3.0.0 (Laake, 2013) as a wrapper for program MARK version 9.0 to estimate the influence of patch‐ and landscape‐scale matrix treatments on the survival and movement of individuals while accounting for imperfect detection.

**FIGURE 2:**
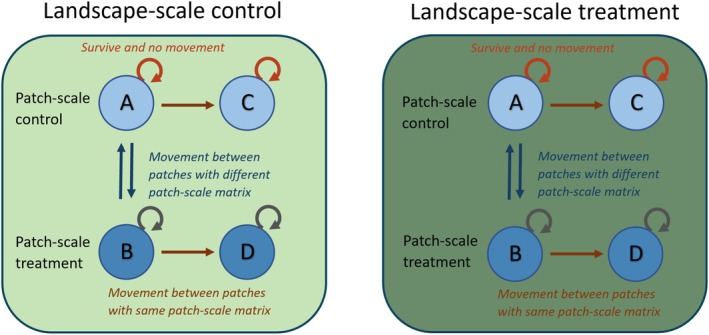
Schematic of the multistate mark recapture model (Lebreton et al., 2009) showing possible transitions between patch‐scale conditions with straight arrows. Circular arrows indicate the possibility of survival with no movement. The survival for individuals was fixed to a single value for patch‐scale control (orange circle arrows) and patch‐scale treatment (grey circle arrows) matrix.

Multi‐state models can be challenging to fit where there are dozens of potential states (e.g. patches), as was the case in this experiment. To simplify this modelling to allow parameter identifiability (Bailey et al., 2010), we collapsed movement information into four possible states based on the movement history of an individual between patches within landscape. These states allow for a focus on directly testing effects for patch‐ and landscape‐scale treatments on movement and survival. Within a landscape, states included: residing in a patch with patch‐scale control matrix (A), residing in a patch with patch‐scale treatment matrix (B), residing in a patch with the same patch‐scale matrix after movement (from A) for patch‐scale control (C), and residing in a patch with same patch‐scale matrix after movement (from B) for patch‐scale treatments (D: see Figure 2). These states allowed for the modelling movement between versus within the two patch‐scale matrix types (treatment v control). We constrained possible transitions to only include movements between control and treatment patches (A → B and B → A), movements from control to control (A → C), and movements from treatment to treatment (B → D). States C and D were considered absorbing states, so the transitions starting from C or D (i.e. C → A, C → B, C → D, D → A, D → B, D → C) were not possible and thus were fixed to zero in the model. Estimated survival in this model was fixed to be the same for both patch‐scale control matrix states (S_A_ = S_C_) and both patch‐scale treatment matrix states (S_B_ = S_D_), since individuals that dispersed within a single patch‐scale matrix type experienced the same patches as those that did not move and those that moved between matrix types.

We constructed a model set that included covariates of the landscape‐ and patch‐scale matrix treatments on survival and on the transition probabilities between states. Both survival and transition probabilities were estimated on a weekly timescale. All possible combinations, including interactions for these two treatments were included in models for both parameters. All models treated detection as a nuisance variable, which was allowed to vary by survey to account for temporal variation. We used model selection with AICc (Anderson & Burnham, 2004), employing 95% CIs to assess the importance of variables in the top model.

## 3 RESULTS

Over the 11 surveys conducted from 2019 to 2021 across the 12 landscapes (5160 patch observations), we relocated 43 originally released individuals, marked 926 new adult *C. vittiger*, and had 170 re‐sights of individuals. In total, we counted 1159 adults and 4769 nymphs across all population surveys, which covered approximately seven generations of *C. vittiger* populations.

### 3.1 Relative importance of matrix scale and interactive effects

**FIGURE 3:**
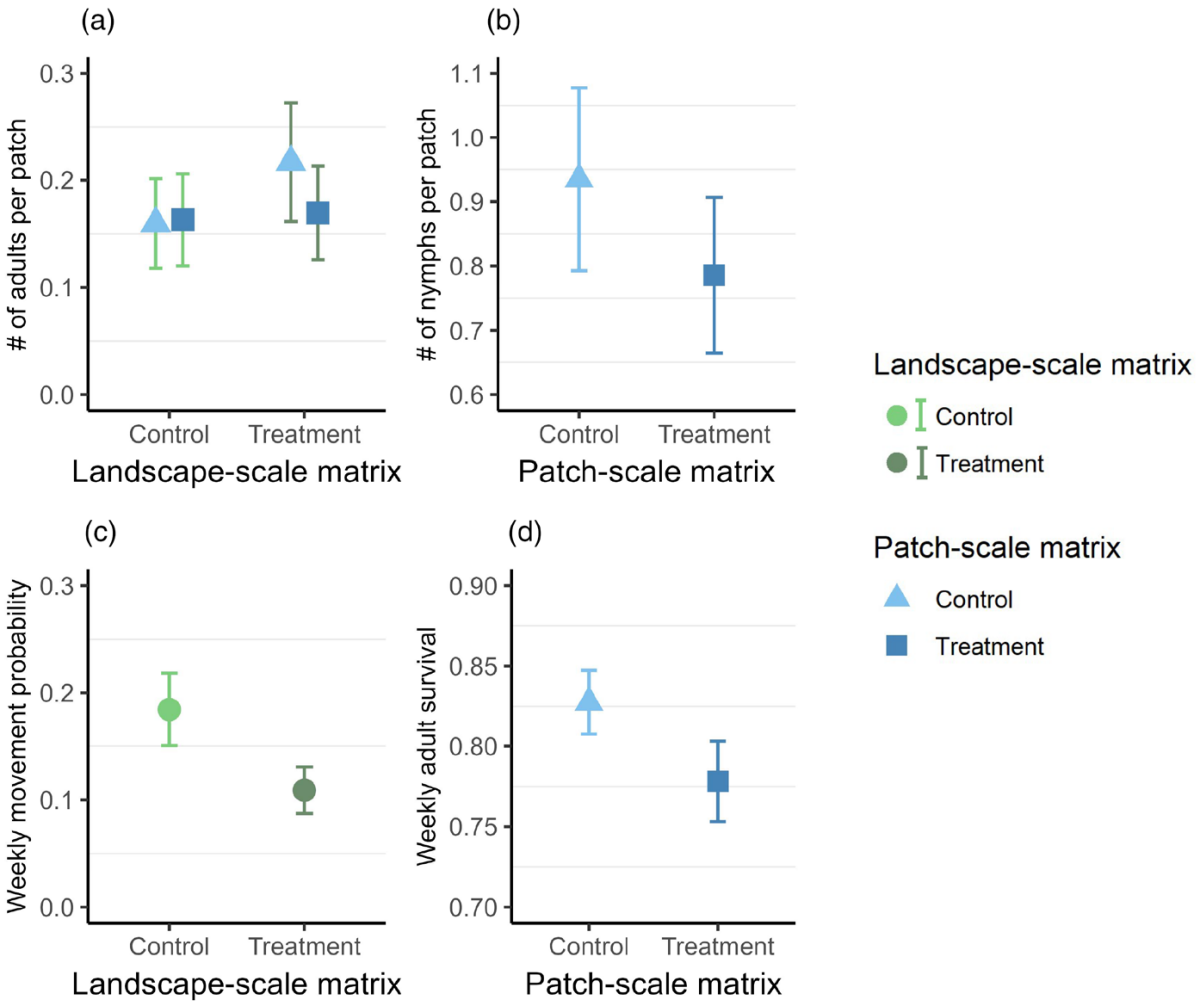
The effects of matrix scale on the abundance, survival, and movement of *C. vittiger*. Green shading indicates the landscape‐scale and blue shading the patch‐scale matrix effects. Uncertainty for parameter estimates given as standard errors. (a) Adult per‐patch abundance showing an interaction with the landscape‐ and patch‐scale matrix. (b) Nymph per‐patch abundances which differed due to patch‐scale matrix effects. (c) Probability of movement for individual *C. vittiger* between patches depending only on the landscape‐scale matrix from the same multi‐state mark–recapture model. (d) Weekly adult survival rates from the top multi‐state mark–recapture model including only patch‐scale effects on survival.

Per‐patch abundance of *C. vittiger* adults was affected by both patch‐ and landscape‐scale matrix treatments and their interaction (Table S1). The experimental reduction of height of the matrix (treatments) co‐occurring at the landscape scale and the patch scale created a negative interactive effect on per‐patch abundance for adult *C. vittiger* (*β*_patch:landscape_ = −0.26; CI_95%_: −0.58, 0.04; Figure 3a). Unmanipulated landscapes (landscape‐scale control) showed no difference in per‐patch abundance based on patch‐scale treatments (Rate Ratio Patch‐scale treatment/control: 1.02; CI_95%_: 0.78, 1.33). In landscapes with reduced matrix quality (landscape‐scale treatments), the patch‐scale treatment led to a decrease in per‐patch abundance compared to patch‐scale control (Rate Ratio Patch‐scale treatment/control: 0.78; CI_95%_: 0.64, 0.96). However, the landscape‐scale treatment did not alter the abundance of *C. vittiger* adults when considered independently (i.e. ignoring the interaction). When comparing only patches that have patch‐scale control matrix (Rate Ratio Landscape‐scale treatment/control: 1.36; CI_95%_: 0.89, 2.07) or only patch‐scale treatment matrix (Rate Ratio Landscape‐scale treatment/control: 1.04; CI_95%_: 0.66, 1.63) the abundances did not differ.

Relative reproductive output of adults as measured by nymph patch abundance was affected only by patch‐scale treatments (Table S5). The patch‐scale treatment decreased the number of nymphs per patch compared to patch‐scale control (*β*_patch‐treatment_ = −0.17; CI_95%_ −0.37, 0.02; Figure 3b).

### 3.2 Disentangling movement and survival processes

Based on the multi‐state mark recapture analysis, survival of individuals was affected by the patch‐scale matrix only. Adult survival increased in control patches relative to treated patches (*β*_patch‐treatment_ = −0.31; CI_95%_ −0.60, −0.03; Figure 3d). Movements between patches were similar across patch‐scale treatments, yet there was a weak tendency for individuals to move less between patches in landscape‐scale treatments in comparison with controls (*β*_landscape‐treatment_ = −0.74; CI_95%_ −1.50, 0.02; Figure 3c). There were four additional models within 2 ΔAICc of the top model presented above (Table S4), yet in each of these additional models, the 95% CI for multiple covariates greatly overlapped zero.

## 4 DISCUSSION

Matrix effects are ubiquitous and yet the multi‐scale mechanisms generating them remain largely unresolved (Driscoll et al., 2013; Fletcher et al., 2024). Our experiment revealed that variation in the matrix at both the patch‐ and landscape‐scales influenced populations in fragmented landscapes, but also that—across demographic rates—the patch‐scale effects were more prevalent. In the case of reproduction (i.e. juvenile abundance) and adult survival, only the patch‐scale matrix was important. In contrast, low matrix quality in a landscape only reduced inter‐patch movement (as expected), but this led to stronger differences from local patch‐scale matrix conditions for adult abundance. Surprisingly, due to this interaction, patches with high‐quality local matrix appear to benefit from reducing the quality of the landscape matrix. Our results suggest that understanding matrix effects requires consideration of the local matrix conditions as well as the landscape matrix context in which those local patch‐scale effects are embedded.

### 4.1 Scale and pattern of population responses

The interaction between patch‐ and landscape‐scale matrix for change in adult abundances indicates that both scales impact a population's response to matrix change. Patch‐scale matrix that was experimentally reduced to ‘low‐quality’ showed the expected decrease in adult abundance, but this only occurred in landscapes that also had reduced landscape matrix. This context‐dependent effect of the matrix directly surrounding habitat co‐occurs with the decreased movements in the landscape with experimentally reduced matrix. Collectively, this suggests that local matrix context is important when the landscape matrix decreases opportunities to transfer between patches of varying quality. We observed a 28% decrease in adult population sizes due to the patch‐scale matrix effects within manipulated landscapes, which is comparable to the 36% increase associated with the landscape matrix treatment for patches with high‐quality control local matrix. This is despite the vastly different areas being manipulated: the patch‐scale treatments manipulated approximately 3% of the landscape whereas the landscape‐scale treatments manipulated approximately 93%. The similar magnitudes of matrix effects despite the large difference in area indicates that lands directly surrounding habitat can have an outsized influence compared with more distant landscape features.

Variation in edge effects is likely driving the patch‐scale effects of the matrix through decreases in patch quality, which were reflected in the reduced survival for individuals and decreased nymphal output in these patches. Surprisingly, the landscape matrix treatment did not have a significant effect on either adult survival or nymph abundance, despite previous observations suggesting a relationship between survival and the average matrix height across a landscape (Fletcher et al., 2018, unpublished data). This previous study did not separate the matrix directly surrounding patches from the broader landscape, and this experiment indicates that these patterns of survival with the landscape matrix are likely being caused by local matrix processes rather than matrix structure at a broader scale.

While we did not measure reproduction directly (e.g. eggs), previous experiments in this system have shown that adult abundance and rate of immigration to a patch can accurately predict the future abundance of nymphs. Moreover, the number of nymphs at a patch can also be used to predict the future abundance of adults (Fletcher et al., 2018). Both time frames used coincided with the development time of *C. vittiger* nymphs, suggesting that abundance is a useful proxy for reproductive output and adult recruitment.

Although immigration and emigration into and out of patches have previously shown to be affected by local matrix type (Haynes, Diekötter, & Crist, 2007; Haynes, Dillemuth, et al., 2007), inter‐patch movements in our landscapes were affected only by landscape‐scale variation in the matrix. In this experiment, we only manipulated the height of the matrix rather than changing land cover type (i.e. switching from vegetation to bare ground); such changes in cover type could be more important for patch immigration or emigration processes than our experimental manipulations (cf. Haynes & Cronin, 2006). For instance, Jonsen and Taylor (2000) found that emigration rates of damselflies did not differ between fully forested and partially forested matrix but did for non‐forested pasture. Surprisingly, the local matrix appeared to have little effect on population size in the high‐quality control landscape matrix. The obscuring of patch‐scale effects by the quality of the landscape matrix provides a potential reason why meta‐analyses of edge effects can fail to find impacts of the local matrix (Magura et al., 2017) and may explain the absence of patch‐scale effects in some matrix experiments which only focus on the local scale (Fletcher et al., 2024). Ultimately, the interactive effects of the matrix between scales arose because of the ability of individuals to move within a landscape and thus change the local patch conditions an individual experiences.

### 4.2 Demographic and movement responses

Although it appears contradictory to theory that in this experiment the more permeable landscape matrix controls resulted in lower overall population size, previous research shows that increasing permeability can increase population extinction and reduced population sizes through spatial synchrony (Vandermeer & Carvajal, 2001), leaking emigrants (Cronin, 2007) and increased effects of movement mortality (Yamaura et al., 2022). Spatial synchronization of population dynamics due to dispersal has been well‐documented (reviewed in Liebhold et al., 2004) and may explain, in part, the absence of patch‐scale matrix effects in our landscapes with high‐quality control matrix. Furthermore, a matrix that permits high movement rates has been shown to increase extinction risk compared to intermediate movement rates, due to the negative risk involved in dispersal (Revilla & Wiegand, 2008) in relation to positive ‘spreading of risk’ effects. High dispersal rates can decrease population size if movement through the matrix is substantially risky (Aars et al., 1999), and in highly mobile species, dispersal mortality can be a dominant demographic process (Schtickzelle et al., 2006). Moreover, theory indicates that intermediate dispersal rates can enhance total population size (relative to very low or high dispersal rates), because it permits some asynchrony in local population dynamics across a metapopulation (Roy et al., 2005).

Simulations by Yamaura et al. (2022) indicate that increasing matrix permeability decreases population vital rates if there is not a corresponding increase in survival while moving through the matrix. Within our empirical system, inclusion of dispersal mortality has been shown to help explain variation in movement and population abundance (Fletcher et al., 2019). The absence of an effect of the landscape‐scale matrix treatment on overall survival in these experimental landscapes suggests that there was not an appreciable and offsetting increase in movement survival. Our results indicate that both movement permeability and movement survival need to be considered independently in evaluation of landscape‐scale matrix quality effects for populations in fragmented systems.

The dependence of movement on only the landscape matrix contrasts with the idea that patch‐scale permeability is responsible for the movement decisions of individuals in fragmented landscapes (Ewers & Didham, 2006; Haynes & Cronin, 2006). In our system, landscape resistance increases in relation to vegetation height of the matrix (see Fletcher et al., 2019), but how individuals perceive matrix height in initial emigration decisions is not yet understood. Matrix type may be more important than simple differences in matrix height in leading to decisions to emigrate from habitat patches (Haynes & Cronin, 2006).

### 4.3 Implications for managing the matrix

The matrix has been highlighted as an area of conservation and restoration concern (Franklin & Lindenmayer, 2009). The outsized influence of the local matrix immediately surrounding patches in this experiment highlights that conservation targeted in areas directly surrounding remaining habitat may be more beneficial than similar amounts of effort targeting entire landscapes. Although increasing connectivity is often a conservation goal (Keeley et al., 2021), benefits from increased movements in a landscape may be offset by low‐quality patches. Both at the local and landscape scale, this experiment indicates that restoration of non‐habitat in landscapes can be implemented to increase the quality and effectiveness of remaining habitat in fragmented landscapes.

While landscape experiments are valuable as they can provide causal inference and control for many confounding factors, they necessarily occur at a small scale relative to many types of human‐induced matrix change (Wiersma, 2022). Our field experiment on a wild population of a specialist species that was designed at a scale relevant to the species' biology shares many patterns observed with other specialist species inhabiting human‐dominated landscapes (Fletcher et al., 2011; Fletcher, Maxwell, et al., 2013). Consequently, carefully designed field experiments can provide insights for human‐dominated landscapes that are driven the similar underlying processes (Wiens et al., 1993; Wiens & Milne, 1989).

## 5 CONCLUSIONS

Our results emphasize that disentangling the effects of local matrix conditions from the broader landscape matrix is essential to understand how non‐habitat in fragmented landscapes affects populations. As conservation efforts increasingly focus on improving landscapes in human‐dominated areas (e.g. Kremen & Merenlender, 2018), our results suggest that one should both modulate local matrix quality to enhance demographic responses, while at the same time improve the landscape‐scale matrix to increase dispersal. Future work should determine whether species responses to these two scales can be predicted by species traits and attributes of variation in land use.

## AUTHOR CONTRIBUTIONS

Thomas A. H. Smith and Robert J. Fletcher conceived of ideas, designed methodology and analysed the data. Thomas A. H. Smith collected the data. Thomas A. H. Smith wrote the initial draft of the manuscript and all authors contributed equally to revisions of the manuscript.

## CONFLICT OF INTEREST STATEMENT

The authors have no conflicts of interest.

## Supporting information


**Table S1:** Model selection table for the per‐patch abundance of *Chelinidea vittiger* adults using a Zero‐Inflated Poisson GLMM with the random terms for the year, landscape, and blocking pair.
**Table S2:** Model estimates from top model for adult abundances at individual patches including the terms for patch and landscape treatments with their interaction as fixed effects.
**Table S3:** Evaluation of model fit using simulated residuals via package DHARMa for the top ZIP model for adult abundance presented in Table S2.
**Table S4:** Model selection table for the per‐patch nymph abundance of *C. vittiger* using a Zero‐Inflated Negative Binomial GLMM with the random terms for year, landscape, and blocking pair.
**Table S5:** Model estimates from top model for nymph abundances at individual patches including the term for patches only as fixed effects.
**Table S6:** Evaluation of model fit using simulated residuals via package DHARMa for the top ZINB model for nymph abundance presented in Table S5.
**Table S7:** Model selection table for multistate mark recapture model (Lebreton et al., 2009) showing the covariates for survival (S) and transition probability between states (Psi).
**Table S6:** Model estimates for the multistate mark recapture model for the covariates for survival (S) and transition probability between states (Psi).
**Figure S1:** Correlation between the number of cactus pads on an individual cactus patch and the basal area (cm^2^) calculated as an ellipse using the major and minor axes.
**Figure S2:** Histrograms showing the distribution of distances between patches using the spatial distribution for all 6 experimental blocks with (a) displaying all the pairwise distances that occur within the landscapes and (b) showing the nearest neighbor distance for every patch in all landscapes.
**Figure S3:** Dot plot showing the distribution of counts for adults across all surveys.
**Figure S4:** Dot plot showing the distribution of counts for nymphs across all surveys.
**Figure S5:** Estimates for detection (p) across all time periods in the multi‐state mark recapture model.

## Data Availability

Data available from the Dryad Digital Repository https://doi.org/10.5061/dryad.kprr4xhh6 (Smith et al., 2025).
