# Supplementary material for: Isolating the role of the matrix at patch and landscape scales
Source: J Anim Ecol. 2025 Jun 24;94(9):1800–10. doi: 10.1111/1365-2656.70089 (PMC12424283; doi:10.1111/1365-2656.70089)
Supplement: Supplementary file 1 — Table S1: Model selection table for the per‐patch abundance of Chelinidea vittiger adults using a Zero‐Inflated Poisson GLMM with the random terms for the year, landscape, and blocking pair. Table S2: Model estimates from top model for adult abundances at individual patches including the terms for patch and landscape treatments with their interaction as fixed effects. Table S3: Evaluation of model fit using simulated residuals via package DHARMa for the top ZIP model for adult abundance presented in Table S2. Table S4: Model selection table for the per‐patch nymph abundance of C. vittiger using a Zero‐Inflated Negative Binomial GLMM with the random terms for year, landscape, and blocking pair. Table S5: Model estimates from top model for nymph abundances at individual patches including the term for patches only as fixed effects. Table S6: Evaluation of model fit using simulated residuals via package DHARMa for the top ZINB model for nymph abundance presented in Table S5. Table S7: Model selection table for multistate mark recapture model (Lebreton et al., 2009) showing the covariates for survival (S) and transition probability between states (Psi). Table S6: Model estimates for the multistate mark recapture model for the covariates for survival (S) and transition probability between states (Psi). Figure S1: Correlation between the number of cactus pads on an individual cactus patch and the basal area (cm2) calculated as an ellipse using the major and minor axes. Figure S2: Histrograms showing the distribution of distances between patches using the spatial distribution for all 6 experimental blocks with (a) displaying all the pairwise distances that occur within the landscapes and (b) showing the nearest neighbor distance for every patch in all landscapes. Figure S3: Dot plot showing the distribution of counts for adults across all surveys. Figure S4: Dot plot showing the distribution of counts for nymphs across all surveys. Figure S5: Estimates for detection (p) acr [file JANE-94-1800-s001.docx]

**Appendix S1**

for

**Isolating the role of the matrix at patch and landscape scale**

***Journal of Animal Ecology***

**Supplementary Tables:**

Table S1. Model selection table for the per-patch abundance of *Chelinidea vittiger* adults using a Zero-Inflated Poisson GLMM with the random terms for the year, landscape, and blocking pair.

| Model | df | Log Likelihood | AICc | ∆AIC | Weight |
| --- | --- | --- | --- | --- | --- |
| Patch × Landscape | 8 | -2,679.6 | 5,414.7 | 0 | 0.35 |
| Patch | 7 | -2,681.7 | 5,416.0 | 0.11 | 0.33 |
| Patch + Landscape | 6 | -2,681.0 | 5,416.8 | 0.84 | 0.23 |
| Intercept | 5 | -2,684.5 | 5,421.6 | 3.90 | 0.05 |
| Landscape | 6 | -2,684.0 | 5,422.5 | 4.81 | 0.03 |

Table S2. Model estimates from top model for adult abundances at individual patches including the terms for patch and landscape treatments with their interaction as fixed effects.

| Fixed Effects | Estimate | Std. Error | P-value |
| --- | --- | --- | --- |
| Intercept | -0.112 | 0.264 | 0.672 |
| Patch | 0.021 | 0.135 | 0.878 |
| Landscape | 0.306 | 0.169 | 0.070 |
| Patch × Landscape | -0.267 | 0.159 | 0.092 |
|  |  |  |  |
| Random Effects | Variance | Std. Dev. |  |
| Block | 0.155 | 0.390 |  |
| Landscape | 0.045 | 0.212 |  |
| Survey Year | 0.079 | 0.281 |  |
|  |  |  |  |
| Zero-Inflation | Estimate | Std. Error | P-value |
| Intercept | 1.526 | 0.059 | <0.001 |
|  |  |  |  |

Table S3. Evaluation of model fit using simulated residuals via package DHARMa for the top ZIP model for adult abundance presented in Table S2.

| Residual Test | Statistic | P-value |
| --- | --- | --- |
| Dispersion (Std. Dev.) | 1.91 | 0.056 |
| Normality (Kolmogorov-Smirnov) | 0.015 | 0.231 |
| Outlier (Bootstrapped) | -- | 0.340 |
| Temporal Autocorrelation (Durbin-Watson) | 0.778 | 0.936 |
|  |  |  |

Table S4. Model selection table for the per-patch nymph abundance of *C. vittiger* using a Zero-Inflated Negative Binomial GLMM with the random terms for year, landscape, and blocking pair.

| Model | df | Log Likelihood | AICc | ∆AIC | Weight |
| --- | --- | --- | --- | --- | --- |
| Patch | 5 | -5,031.9 | 10,073.8 | 0 | 0.39 |
| Intercept | 6 | -5,031.6 | 10,075.1 | 0.95 | 0.24 |
| Patch + Landscape | 7 | -5,031.1 | 10,076.1 | 1.68 | 0.17 |
| Patch × Landscape | 4 | -5,037.8 | 10,083.5 | 2.64 | 0.10 |
| Landscape | 5 | -5,037.5 | 10,085.0 | 2.84 | 0.09 |

Table S5. Model estimates from top model for nymph abundances at individual patches including the term for patches only as fixed effects.

| Fixed Effects | Estimate | Std. Error | P-value |
| --- | --- | --- | --- |
| Intercept | 1.66 | 0.164 | <0.001 |
| Patch | -0.17 | 0.10 | 0.087 |
|  |  |  |  |
| Random Effects | Variance | Std. Dev. |  |
| Block | 0.080 | 0.283 |  |
| Landscape | <0.001 | 0.0002 |  |
| Survey Year | 0.012 | 0.111 |  |
|  |  |  |  |
| Zero-Inflation | Estimate | Std. Error | P-value |
| Intercept | 1.53 | 0.086 | <0.001 |

Table S6. Evaluation of model fit using simulated residuals via package DHARMa for the top ZINB model for nymph abundance presented in Table S5.

| Residual Test | Statistic | P-value |
| --- | --- | --- |
| Dispersion (Std. Dev.) | 0.95 | 0.968 |
| Normality (Kolmogorov-Smirnov) | 0.01 | 0.779 |
| Outlier (Bootstrapped) | -- | 0.560 |
| Temporal Autocorrelation (Durbin-Watson) | 1.81 | 0.742 |
|  |  |  |

Table S7. Model selection table for multistate mark recapture model (Lebreton et al. 2009) showing the covariates for survival (S) and transition probability between states (Psi). All models included survey period as a covariate on detection.

| S | Psi | df | Log Likelihood | AICc | ∆AIC | Weight |
| --- | --- | --- | --- | --- | --- | --- |
| Patch | Landscape | 12 | -446.1 | 916.6 | 0.00 | 0.17 |
| Patch + Landscape | Landscape | 13 | -445.3 | 917.1 | 0.50 | 0.13 |
| Patch × Landscape | Landscape | 14 | -444.8 | 918.0 | 1.47 | 0.08 |
| Patch | Intercept | 11 | -448.0 | 918.2 | 1.62 | 0.08 |
| Patch | Patch + Landscape | 13 | -446.1 | 918.5 | 1.97 | 0.06 |
| Patch + Landscape | Intercept | 12 | -447.2 | 918.7 | 2.12 | 0.06 |
| Patch + Landscape | Patch + Landscape | 14 | -445.3 | 919.0 | 2.48 | 0.05 |
| Intercept | Landscape | 11 | -448.5 | 919.2 | 2.71 | 0.04 |
| Patch × Landscape | Intercept | 13 | -446.6 | 919.6 | 3.05 | 0.04 |
| Patch × Landscape | Patch + Landscape | 15 | -444.8 | 920.0 | 3.45 | 0.03 |
| Patch | Patch | 12 | -448.0 | 920.2 | 3.66 | 0.03 |
| Landscape | Landscape | 12 | -448.0 | 920.3 | 3.73 | 0.03 |
| Patch | Patch × Landscape | 14 | -446.0 | 920.6 | 4.01 | 0.02 |
| Patch + Landscape | Patch | 13 | -447.2 | 920.7 | 4.16 | 0.02 |
| Intercept | Intercept | 10 | -450.3 | 920.9 | 4.34 | 0.02 |
| Patch + Landscape | Patch × Landscape | 15 | -445.3 | 921.0 | 4.51 | 0.02 |
| Intercept | Patch + Landscape | 12 | -448.5 | 921.3 | 4.72 | 0.02 |
| Patch × Landscape | Patch | 14 | -446.6 | 921.7 | 5.09 | 0.01 |
| Landscape | Intercept | 11 | -449.8 | 921.9 | 5.36 | 0.01 |
| Patch × Landscape | Patch × Landscape | 16 | -444.8 | 922.0 | 5.49 | 0.01 |
| Landscape | Patch + Landscape | 13 | -448.0 | 922.3 | 5.74 | 0.01 |
| Intercept | Patch | 11 | -450.3 | 922.9 | 6.38 | 0.01 |
| Intercept | Patch × Landscape | 13 | -448.5 | 923.3 | 6.73 | <0.01 |
| Landscape | Patch | 12 | -449.8 | 924.0 | 7.41 | <0.01 |
| Landscape | Patch × Landscape | 14 | -447.8 | 924.3 | 7.77 | <0.01 |

Table S6. Model estimates for the multistate mark recapture model for the covariates for survival (S) and transition probability between states (Psi). All models included survey period as a covariate on detection.

| Weekly Survival (S) | Estimate | Std. Error | Lower 95% CI | Upper 95% CI |  |
| --- | --- | --- | --- | --- | --- |
| Patch-scale Control | 0.827 | 0.020 | 0.785 | 0.863 |  |
| Patch-scale Treatment | 0.778 | 0.025 | 0.725 | 0.823 |  |
|  |  |  |  |  |  |
| Weekly Movement (Psi) | | Estimate | Std. Error | Lower 95% CI | Upper 95% CI |
| Landscape Control | | 0.185 | 0.034 | 0.127 | 0.260 |
| Landscape Treatment | | 0.109 | 0.022 | 0.073 | 0.159 |

**
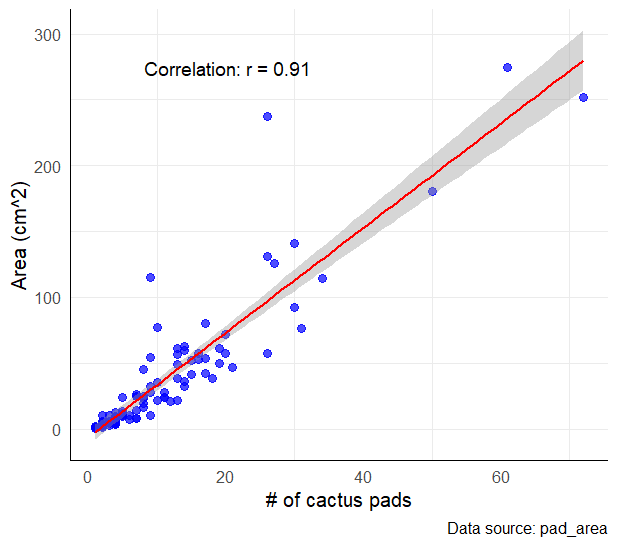
**

Figure S1: Correlation between the number of cactus pads on an individual cactus patch and the basal area (cm^2^) calculated as an ellipse using the major and minor axes. Numbers were measured from 100 randomly sampled wild patches at Ordway Swisher Biological Station in 2018.

**
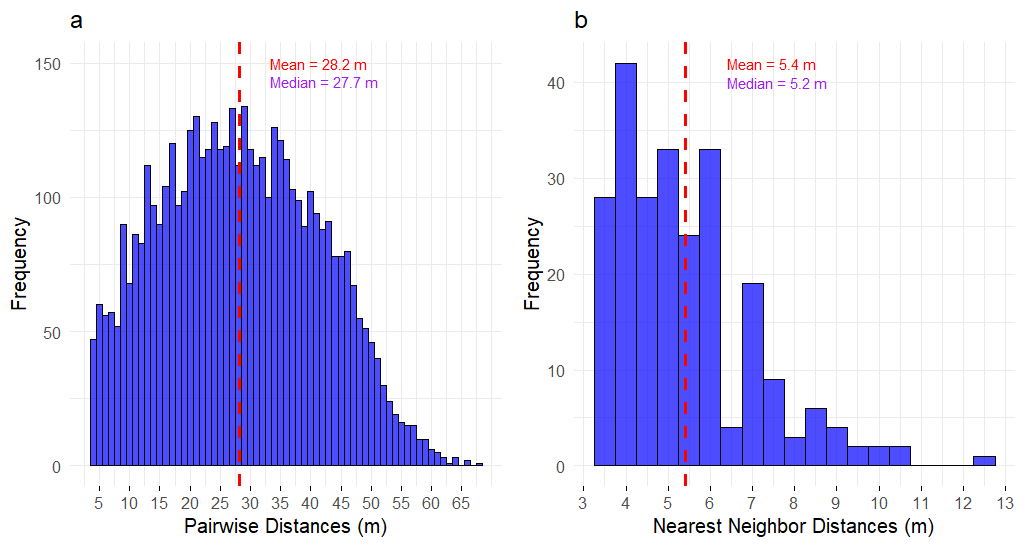
**

Figure S2: Histrograms showing the distribution of distances between patches using the spatial distribution for all 6 experimental blocks with (a) displaying all the pairwise distances that occur within the landscapes and (b) showing the nearest neighbor distance for every patch in all landscapes.


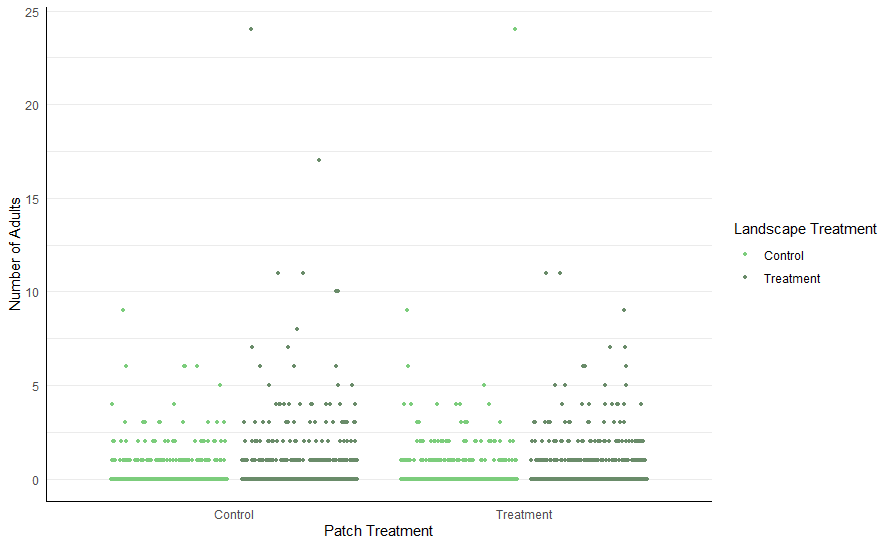


Figure S3: Dot plot showing the distribution of counts for adults across all surveys. The x-axis indicates the patch-scale matrix, and the colors represent the landscape-scale matrix.


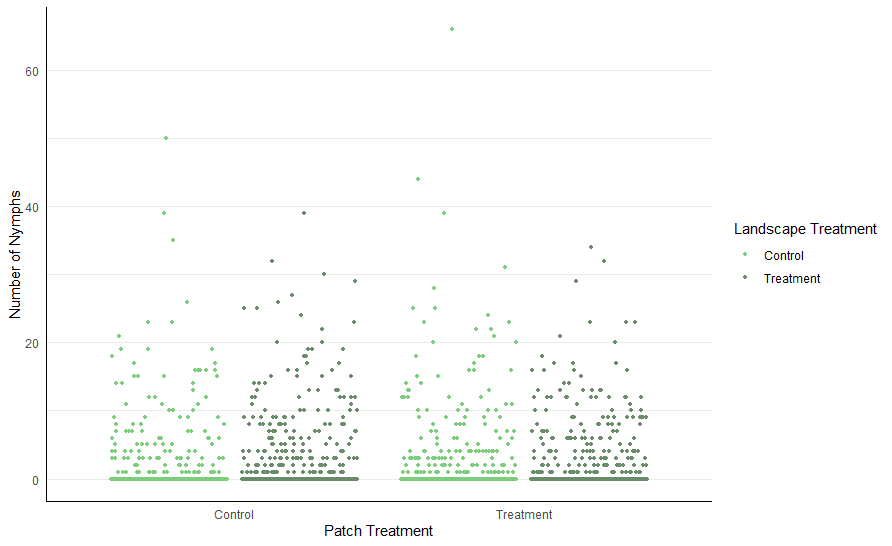
Figure S4: Dot plot showing the distribution of counts for nymphs across all surveys. The x-axis indicates the patch-scale matrix, and the colors represent the landscape-scale matrix.


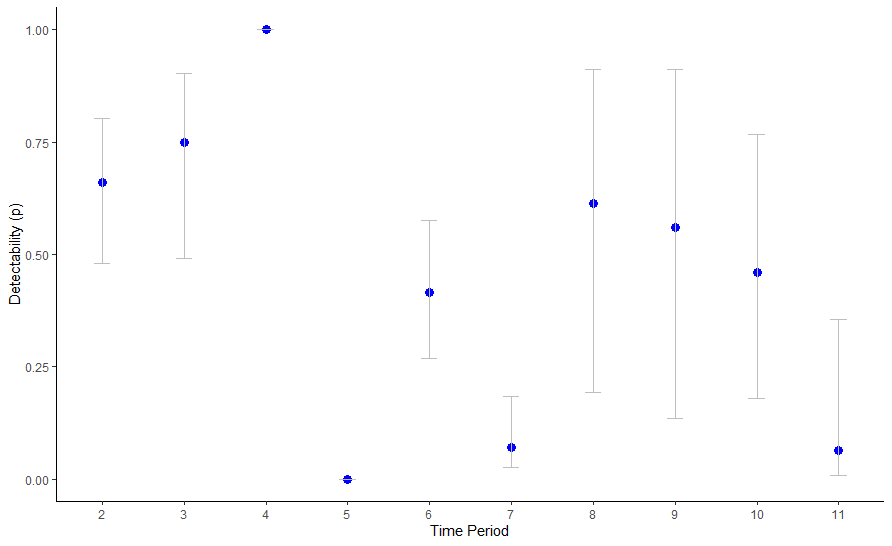


Figure S5: Estimates for detection (p) across all time periods in the multi-state mark recapture model. The error bars indicate 95% confidence intervals.
